# Are all ventilators for NIV performing the same? A bench analysis

**DOI:** 10.1007/s10877-023-01019-z

**Published:** 2023-07-31

**Authors:** Sara Martínez-Castro, Francisco Javier Belda Nacher, Jaume Puig Bernabeu, Marina B Soro Domingo, Carlos Delgado Navarro, Héctor Ortega Pons

**Affiliations:** 1 Anesthesia and Critical Care Department, Hospital Clínico Universitario de Valencia (HCUV), Valencia, Spain; 2 Universidad de Valencia (UV), Valencia, Spain; 3 Anesthesia and Critical Care Department, Consorcio Hospital General Universitario de Valencia (CHGUV), Valencia, Spain; 4 Instituto de Investigación Sanitaria de Valencia (INCLIVA), Valencia, Spain; 5 Anesthesia Department, Hospital IMED Valencia (ES), IMED, Valencia, Spain

**Keywords:** Bench study, Breathing mechanics, Computer simulation, Noninvasive ventilation, Respiratory mechanics, Ventilator performance

## Abstract

Global pandemic due to COVID-19 has increased the interest for ventilators´ use worldwide. New devices have been developed and older ones have undergone a renewed interest, but we lack robust evidence about performance of each ventilator to match appropriate device to a given patient and care environment. The aim of this bench study was to investigate the performance of six devices for noninvasive ventilation, and to compare them in terms of volume delivered, trigger response, pressurization capacity and synchronization in volume assisted controlled and pressure support ventilation. All ventilators were tested under thirty-six experimental conditions by using the lung model ASL5000® (IngMar Medical, Pittsburgh, PA). Two leak levels, two muscle inspiratory efforts and three mechanical patterns were combined for simulation. Trigger function was assessed by measurement of trigger-delay time. Pressurization capacity was evaluated as area under the pressure–time curve over the first 500 ms after inspiratory effort onset. Synchronization was evaluated by the asynchrony index and by incidence and type of asynchronies in each condition. All ventilators showed a good performance, even if pressurization capacity was worse than expected. Leak level did not affect their function. Differences were found during low muscle effort and obstructive pattern. In general, Philips Trilogy Evo/EV300 and Hamilton C3 showed the best results. NIV devices successfully compensate air leaks but still underperform with low muscle effort and obstructive lungs. Clinicians´ must have a clear understanding of the goals of NIV both for devices´ choice and set main parameters to achieve therapy success.

**Supplementary Information:**

The online version contains supplementary material available at 10.1007/s10877-023-01019-z.

## Introduction

The leading role that advanced hemodynamic or neurological monitoring has taken in recent years should not overshadow the ubiquitous ventilators. Pandemic due to COVID-19 has enhanced the role of these devices in critical care units, around other hospital areas and for home care. By following a recent review and as a result of the daily work with patients we could establish the main goals of ventilation as (1) do not harm, (2) ensure adequate gas exchange and (3) promote patient comfort [1]. These are the objectives that any ventilator should have, so clinicians in charge must know how ventilators work, which setting options they give and if their informative screens offer the whole truth. But few studies systematically analyze devices´ performance, so actually decisions can be solely based on theoretical principles.

Thille et al. in 2009 [2] stated that maybe we had reached the ceiling of technological development in terms of ventilators development. But while new devices continue to reach the market, bench studies that provide information about performance characteristics must be developed. The results of these works can support clinicians´ decisions in terms of indications and adjustments based on patient mechanical characteristics, influence the choice in equipment acquisition and encourage manufacturers to implement improvements in ventilators and their settings. The following study tries to give light to some of the above-mentioned statements.

## Objective

The objective of the present work was to describe the performance of six ventilators during non-invasive ventilation (NIV) in Emergency or Respiratory therapy and Intensive Care Unit (ICU) scenario. The ventilators were assessed under two ventilatory modes: volume assist-control ventilation (ACV) and pressure support ventilation (PSV). Using a lung simulator, thirty-six experimental conditions were created, by the combination of three respiratory mechanical patterns and two leak levels under low and normal muscle effort. With these experimental conditions, the following variables were evaluated: (1) Tidal Volume delivered (V_T_) in ml, (2) Trigger-delay time (TDT) in ms, and (3) Pressurization capacity as pressure–time product in the first 500 ms (PTP500). Even if safety tends to predominate over comfort objectives, ventilation must ensure both of them. As synchronization directly correlates to the comfortable patient-ventilator interaction, our study also analyzed (4) Incidence and type of asynchronies developed.

## Material and methods

### Devices

Six devices suitable for NIV and available in European hospitals were studied: Savina 300 (Dräger, Germany), Elisa 500 (Löwenstein Medical, Germany), Hamilton C3 (Hamilton Medical, Switzerland), Servo Air (Maquet, Sweden), Mindray 300 (Mindray Biomedical, China) and Philips Trilogy Evo/EV300 (Philips, United States of America). They were used with the appropriate limbs and connectors according to each manufacturer guidelines and calibrated before each laboratory session.

### Lung model

For the experiment we used the lung model Active Servo Lung 5000® (ASL5000®- IngMar Medical, Pittsburgh, PA; *software version* SW3.6). It consists of a computer- operated piston that simulates spontaneous breathing by displacing a predetermined volume; piston displacement is controlled following the equation of motion of the Respiratory System and allows adjusting the values of airway resistance (Raw) and compliance of the respiratory system (Crs), mimicking different mechanical conditions and inspiratory muscle efforts. [3, 4] To simulate different leak levels, Simulator Bypass and Leak Valve Module of ASL5000^©^, was also used. This simulator has been used and validated in many previous works [5–14]. Before each session, calibration of pressure, flow and volume was performed against standards using a custom pressure water column, a differential pressure flowmeter (Validyne MP45, ± 2.5 cm H_2_O, Northridge, CA) and a calibration syringe (Hans Rudolph KS, USA).

### Simulated breathing test

#### Respiratory system conditions

For analysis, three mechanical patterns of the respiratory system were defined: standard (S) [Crs = 50 ml/cm H_2_O, Raw = 5 cm H_2_O*s/l], obstructive (O) [Crs = 50, Raw = 20] and restrictive (R) [Crs = 20, Raw = 5]. [15–17].

#### Spontaneous ventilation

Choosing a scheme similar to previous analysis [2, 7–10, 16, 18–26], standard settings were adjusted as spontaneous respiratory rate (RR) 12 breaths per minute (bpm); two levels of inspiratory muscle effort were defined by airway occlusion pressure (P_0.1_): low (Le) P_0.1_ = −0.9 cm H_2_O, and normal (Ne) P_0.1_ = −3.5 cm H_2_O [27–29]. Patient effort model selected was sinusoidal, with inspiratory rise time 15%, inspiratory hold time 0%, inspiratory release time 18.3% and no expiratory activity.

#### Ventilatory modes and settings

Ventilators were set in ACV with V_T_ 500 ml, and in PSV with two levels of pressure (Paw): 10 (PSV10) and 20 (PSV20) cm H_2_O. Ventilator respiratory rate was 10 bpm (I/E 1:2 in ACV), PEEP 5 cm H_2_O with minimal flow triggering (maximum sensitivity) of 0.5–1 lpm without developing auto- triggering (AT). If AT was detected, increase in flow triggering was adjusted up to 2 lpm to avoid them [30]. Higher flow triggering would increase the TDT resulting in a decrease in PTP500; this may worsen patient work of breathing and respiratory drive promoting patient-ventilator asynchronies. Other settings remained by default, including bias flow (set by manufacturer). Two leak levels were also simulated by using the Simulator Bypass and Leak Valve Module of ASL5000^©^: 6 lpm (moderate, M) and 10 lpm (high, H) measured at 10 cm H_2_O [31]. In PSV, the fastest value for pressurization rate (shorter rise time) was set, expiratory trigger at 25% peak flow and other settings by default.

With both ventilatory modes (ACV and PSV) combining mechanical pattern, inspiratory muscle effort and leak level, thirty-six experimental conditions were obtained (see Table 1). In all of them a minimum time of one minute was left for stabilization of the system (until clear sequence of cycles with similar morphology appeared); then consecutive respiratory cycles corresponding to one minute (minimum of 10 cycles) were recorded for subsequent analysis. The curves and data values of muscle pressure (Pmus), airway pressure (Paw) and flow were recorded and exported to an Excel spreadsheet. With those curves delivered volume, trigger response, pressurization capacity and asynchronies (incidence and type) were evaluated; sampling frequency of the curves was 512 Hz.

**Table 1:** Experimental conditions in the bench test

Effort	Leak	Pattern	Ventilatory mode		
			ACV	PSV10	PSV20
Low	Moderate	S	1	13	25
		O	2	14	26
		R	3	15	27
	High	S	4	16	28
		O	5	17	29
		R	6	18	30
Normal	Moderate	S	7	19	31
		O	8	20	32
		R	9	21	33
	High	S	10	22	34
		O	11	23	35
		R	12	24	36

*ACV* assist-control ventilation, *PSV* pressure support ventilation, *S* standard mechanical pattern, *O* obstructive mechanical pattern, *R* restrictive mechanical pattern

#### Measurements

**Fig. 1:**
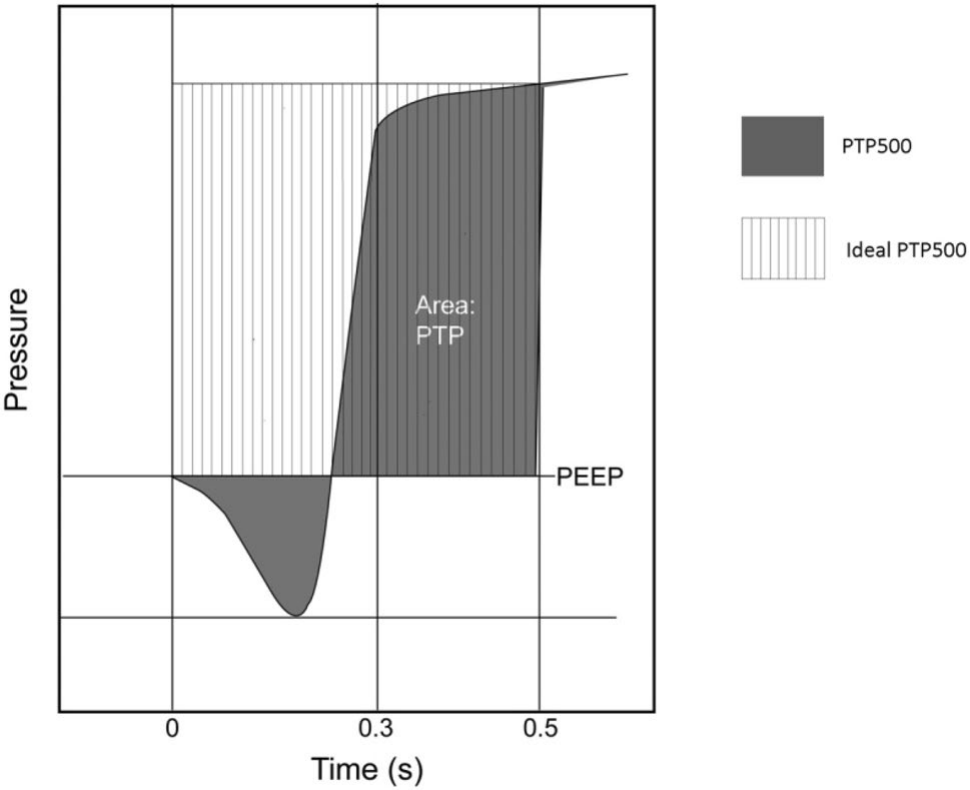
Graphic representation of PTP500 [2]. PTP500: pressure-time product in the first 500 ms. *s* seconds

Delivered V_T_ was measured in ml for all synchronous respiratory cycles in ACV; when synchronization was not achieved in any cycle, V_T_ was measured in auto-triggering (AT) cycles in which V_T_ is theoretically equivalent to the V_T_ delivered in a controlled cycle. Trigger response was evaluated as the delay in triggering response (TDT) in ms, measured from the initial drop in muscular pressure to the onset of inspiratory flow above default bias flow. Pressurization capacity was evaluated through PTP500, in % of ideal Pressure–time product (see Fig. 1) measured as the area under the airway pressure from the initial drop to 500 ms. Synchronization was evaluated by Asynchrony Index (AI)and types of asynchronies (time and flow asynchronies):

(A) Asynchrony Index (AI): is the simplest method to evaluate synchronization. It is calculated as the number of asynchronous events divided by the total number of respiratory cycles (sum of triggered and non-triggered cycles), expressed as a percentage. It takes into account: ineffective efforts (IE), auto-triggering (AT), reverse triggering (RT) and double trigger (DT). An AI ≥ 10% is considered clinically relevant. [3–7].

(B) Qualitative analysis of the asynchronies developed during the experiment: carried out by inspection and visual detection of the flow curves, Paw and Pmus of the recorded cycles. The asynchronies developed in all conditions were mainly time asynchronies (IE, AC and RT). [8].

#### Protocol sequence

Before initiating any ventilator experiment, we assessed P_0.1_ by recording an occlusion maneuver in the inspiratory port of the lung model (see Supplementary Material: Occlusion maneuver, Table 1 and Figs. 1–4 in Supplementary material). After stabilization of the ventilator-lung system, ten to twelve breathing cycles were recorded at each condition and stored for off-line analysis.

#### Statistical analysis

Each parameter value was represented as mean and standard deviation (SD) of ten breaths (whenever it was possible). Standard deviation showed very small differences (range 1–2%) as expected during bench test conditions; but SD was not representative when high incidence of asynchronies appeared. We used Kruskal–Wallis rank sum test to detect statistical significant differences among groups. Once differences among pairs of ventilators were found, we used Wilcoxon rank sum exact test to find out which groups were statistically different. A P value < 0.05 was considered statistically significant. Asynchrony analysis was performed by visual inspection of respiratory cycle graphs for each experimental condition; the evaluation was carried out individually by two researchers on the same traces agreeing on type and magnitude of asynchronies. Values taken for reference were based on the safety standards in design and manufacture of ventilators for home use (ISO 80601-2-72:2015 *Medical electrical equipment*) [9] assuming a negligible variability intra-condition that was not considered clinically relevant [3, 6, 10].

## Results

### Volume delivered

V_T_ was measured in all conditions, but the clinical purposes of this study made it essential to highlight the V_T_ in ACV to assess the accuracy in volume delivered in controlled ventilation conditions (assuming variations up to 10%). Obviously, VT in PSV conditions would vary according to support pressure set for each patient requirements. Table 2 recorded V_T_ in ml and deviation from the 500 ml set (in %). Elisa 500 was not represented because it does not include ACV mode; V_T_ recorded for Servo Air and Mindray 300 were also excluded because they do offer ACV only for invasive ventilation and do not have leak compensation if ACV is used during NIV.

**Table 2:** Mean tidal volume in BTPS delivered by each ventilator in all conditions during ACV, and percentage of deviation from the pre-set 500 ml

ACV	Condition	Dräger Savina 300		Hamilton C3		Philips Trilogy Evo/EV300	
		Mean ± SD	%D	Mean ± SD	% D	Mean ± SD	% D
Low effort							
Moderate Leak	1 S	531 ± 3	6	530 ± 1	6	483 ± 5	−3
	2 O	529 ± 2	6	528 ± 1	6	482 ± 6	−4
	3 R	558 ± 4	12	531 ± 2	6	480 ± 3	−4
Low effort							
High Leak	4 S	528 ± 6	6	539 ± 10	8	487 ± 7	−3
	5 O	516 ± 3	3	528 ± 1	6	475 ± 7	−5
	6 R	526 ± 2	5	530 ± 1	6	486 ± 5	−3
Normal effort							
Moderate Leak	7 S	641 ± 2	28	577 ± 6	15	554 ± 0	11
	8 O	516 ± 3	3	528 ± 1	6	515 ± 1	3
	9 R	529 ± 2	6	527 ± 7	5	503 ± 9	1
Normal effort							
High Leak	10 S	647 ± 3	29	566 ± 6	13	607 ± 1	21
	11 O	523 ± 6	5	520 ± 5	4	479 ± 1	−4
	12 R	527 ± 4	5	532 ± 4	6	500 ± 6	0
Mean ± SD		548 ± 46	10 ± 9%	536 ± 17	7 ± 3%	504 ± 39	1 ± 8%

Condition nomenclature in Table 1. *BTPS* Body Temperature Pressure Saturated, *SD* standard deviation

**Fig. 2:**
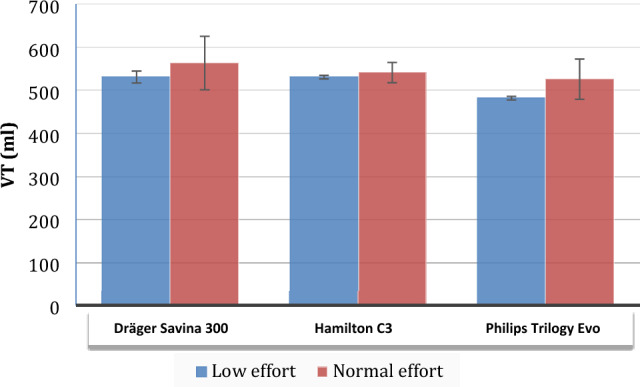
Mean tidal volume delivered in all conditions for the ventilators analyzed in ACV. Differences with low and normal effort

Dräger Savina 300, Hamilton C3 and Philips Trilogy Evo/EV300 delivered the prescribed tidal volume in the expected range (settings ± 10%) for almost all conditions regardless air leak or inspiratory effort. All ventilators tended to deliver higher tidal volumes during normal effort in standard lungs (Fig. 2).

#### General performance

It is remarkable that ACV mode for NIV was only available in three out of the six ventilators analyzed. All of them showed a good performance by assuring volume delivered regardless changing conditions. During standard mechanical pattern, normal inspiratory muscle effort increased V_T_ for the three ventilators (from 11 to 29%).

### Trigger delay time (TDT)

**Fig. 3:**
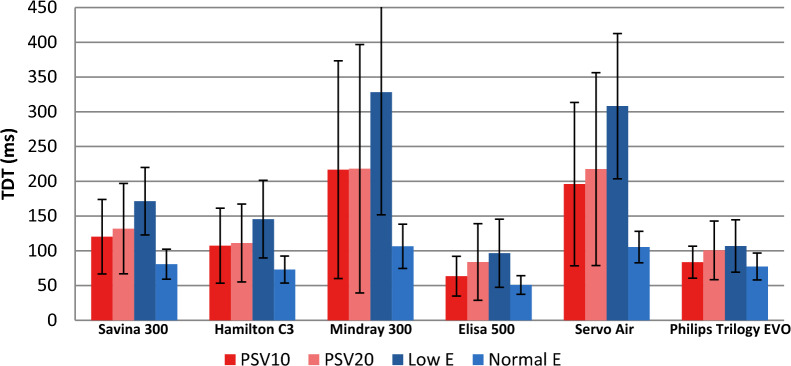
Mean trigger delay time (TDT) (mean and SD) while changes in muscle inspiratory effort and pressure support (values are averaged of the three mechanical patterns and moderate and high leak conditions). *PSV* pressure support ventilation, *E* effort

**Fig. 4:**
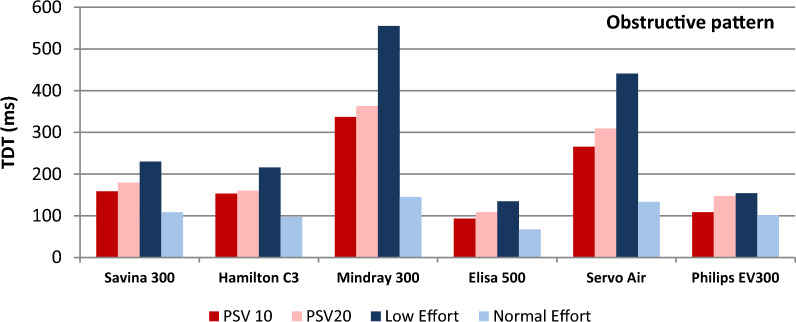
Mean trigger delay time (TDT) under obstructive mechanical pattern while changes in muscle inspiratory effort and pressure support (values are averaged of moderate and high leak conditions). *PSV* pressure support ventilation, *E* effort

TDT was measured in all conditions for the six ventilators analyzed (see complete values in Table 2 in Supplementary Material). It is interesting to evaluate TDT during PSV (Table 3) and pay special attention in obstructive mechanical pattern, as TDT is directly related to comfort during NIV, especially at home, and numerous users of home-care ventilators are patients with obstructive pathology. Values higher than 200 ms have been described as clinical limit related to dyspnea [26, 32]. Table 3 also includes trigger sensitivity adjustment for each ventilator, as it directly influences TDT results. Figure 3 shows TDT in ms for conditions of low and normal effort in PSV; Fig. 4 shows TDT in ms for conditions with obstructive mechanical pattern in PSV.

**Table 3:** Trigger delay time (mean ± SD) recorded from each ventilator during PSV 10 and 20 cm H_2_O

			Dräger Savina 300	Hamilton C3	Mindray 300	Elisa 500	Servo Air	Philips Trilogy Evo
			Trigger sensitivity					
Conditions			1 lpm	1 lpm	0.5 lpm	0.5 lpm	1.6 lpm	1 lpm
			Mean ± SD					
PSV 10	Moderate leak	S	152 ± 14	97 ± 10	203 ± 17	3 ± 3	203 ± 19	74 ± 4
Low effort		O	203 ± 12	225 ± 8	512 ± 49	120 ± 12	401 ± 23	126 ± 7
		R	108 ± 11	115 ± 14	231 ± 14	57 ± 5	241 ± 22	86 ± 5
	High leak	S	146 ± 22	110 ± 7	216 ± 9	56 ± 5	224 ± 17	76 ± 4
		O	226 ± 20	201 ± 15	543 ± 39	123 ± 13	417 ± 36	126 ± 10
		R	132 ± 24	108 ± 12	245 ± 18	56 ± 13	255 ± 10	87 ± 7
PSV 10	Moderate leak	S	68 ± 12	54 ± 4	88 ± 4	44 ± 3	92 ± 10	60 ± 6
Normal effort		O	104 ± 6	93 ± 9	148 ± 19	65 ± 4	126 ± 6	95 ± 6
		R	65 ± 5	65 ± 2	78 ± 12	39 ± 3	92 ± 6	61 ± 5
	High leak	S	70 ± 11	62 ± 7	89 ± 8	33 ± 7	95 ± 7	64 ± 5
		O	103 ± 10	94 ± 4	145 ± 6	66 ± 4	119 ± 9	88 ± 4
		R	68 ± 7	63 ± 3	104 ± 10	52 ± 22	86 ± 7	61 ± 8
PSV 20	Moderate leak	S	144 ± 17	98 ± 8	185 ± 9	212 ± 47	245 ± 17	85 ± 7
Low effort		O	253 ± 15	215 ± 15	583 ± 66	148 ± 18	462 ± 34	181 ± 16
		R	132 ± 13	136 ± 26	219 ± 23	62 ± 3	261 ± 10	86 ± 11
	High leak	S	143 ± 41	104 ± 6	200 ± 20	59 ± 4	236 ± 35	68 ± 13
		O	239 ± 13	223 ± 17	584 ± 50	150 ± 16	486 ± 38	184 ± 15
		R	179 ± 37	114 ± 19	222 ± 11	62 ± 4	266 ± 8	103 ± 9
PSV20	Moderate leak	S	62 ± 7	59 ± 4	69 ± 6	43 ± 1	80 ± 16	61 ± 4
Normal Effort		O	110 ± 5	101 ± 5	151 ± 10	69 ± 6	146 ± 6	110 ± 7
		R	68 ± 6	64 ± 5	98 ± 10	42 ± 5	94 ± 6	74 ± 7
	High leak	S	67 ± 8	54 ± 4	75 ± 8	43 ± 5	99 ± 6	67 ± 5
		O	117 ± 4	103 ± 7	137 ± 10	69 ± 5	145 ± 5	115 ± 8
		R	70 ± 7	64 ± 6	96 ± 9	46 ± 5	91 ± 10	74 ± 8

Mechanical pattern: S (standard), O (obstructive) and R (restrictive)

#### General performance

There were great differences regarding TDT for the analyzed ventilators. Leak level did not affect TDT significantly. Changes in PSV from 10 to 20 cm H_2_O slightly worsened TDT (from 0 to 60 ms, variation around 15%) that could explain in part the pressurization worsening (see next section). In contrast, low effort almost doubled TDT values for most of the ventilators (except for Philips Trilogy Evo/EV300).

Elisa 500 and Philips Trilogy Evo/EV300 showed the best results regardless PSV level, mechanical pattern, leak level and inspiratory effort followed by Hamilton C3 and Dräger Savina 300. Focusing on obstructive mechanical pattern, most common in clinical practice, Elisa 500 and Philips Trilogy Evo/EV300 showed again the best results with TDT < 100 ms in almost all conditions (similar range as ICU ventilators). As TDT is closely related to trigger sensitivity, we tried to improve ventilators´ performance for those with worse TDT results (namely Servo Air and Mindray 300) by precise trigger adjustment. In fact, Mindray 300 trigger sensitivity by default was 2 lpm but we set it at 0.5 lpm (Table 3). Even though, they both showed significantly higher TDT results.

### Pressurization capacity, analyzed as PTP500

PTP500 was measured in PSV conditions for the six ventilators analyzed (Table 4). Pressure values were taken from the beginning of any detected inspiratory effort and up to 0.5 s. At 20 breaths per minute (and I:E 1:2),a time-span of 0.5 s from the beginning of inspiratory effort is 50% of the inspiratory time. We considered that during this time the pressure delivered to the patient should be at least 50% of the ideal pressurization, based on previous publications [32, 33] and our clinical experience, so good pressurization capacity for NIV devices should show PTP500 values over 50%.

**Table 4:** Percentage of Ideal PTP500 (mean percentage ± standard deviation) recorded for each ventilator during PSV10 and PSV20

				Dräger Savina300	Hamilton C3	Mindray 300	Elisa 500	Servo Air	Philips Trilogy Evo
PSV10	Low effort	Moderate leak	S	49 ± 4	69 ± 1	32 ± 3	71 ± 0	34 ± 3	66 ± 1
			O	47 ± 3	48 ± 1	−7 ± 1	66 ± 2	6 ± 4	64 ± 1
			R	62 ± 4	70 ± 3	33 ± 4	77 ± 1	29 ± 1	69 ± 1
		High Leak	S	47 ± 6	69 ± 1	32 ± 3	70 ± 1	30 ± 3	67 ± 0
			O	41 ± 4	47 ± 1	−9 ± 1	64 ± 4	3 ± 3	64 ± 1
			R	57 ± 6	70 ± 2	30 ± 5	78 ± 3	29 ± 2	69 ± 0
	Normal Effort	Moderate leak	S	50 ± 3	56 ± 0	37 ± 1	61 ± 1	48 ± 1	55 ± 0
			O	65 ± 1	71 ± 1	41 ± 1	74 ± 1	52 ± 1	66 ± 0
			R	66 ± 1	76 ± 0	51 ± 1	74 ± 1	59 ± 1	63 ± 1
		High Leak	S	48 ± 5	56 ± 2	37 ± 2	62 ± 0	47 ± 1	55 ± 1
			O	67 ± 3	72 ± 1	41 ± 1	74 ± 1	51 ± 1	66 ± 1
			R	64 ± 3	77 ± 1	45 ± 4	72 ± 4	57 ± 1	63 ± 0
PSV20	Low effort	Moderate leak	S	44 ± 4	42 ± 1	32 ± 2	68 ± 9	25 ± 3	59 ± 1
			O	38 ± 3	47 ± 3	−4 ± 0	60 ± 4	−1 ± 0	49 ± 1
			R	56 ± 4	48 ± 4	31 ± 5	80 ± 1	25 ± 1	62 ± 1
		High Leak	S	43 ± 8	42 ± 1	27 ± 4	68 ± 1	24 ± 2	60 ± 1
			O	41 ± 3	46 ± 3	−4 ± 0	61 ± 3	−2 ± 1	48 ± 1
			R	45 ± 8	52 ± 4	29 ± 3	80 ± 1	24 ± 2	62 ± 1
	Normal Effort	Moderate leak	S	49 ± 1	31 ± 0	29 ± 1	54 ± 0	42 ± 1	54 ± 0
			O	66 ± 1	71 ± 1	46 ± 1	75 ± 1	53 ± 1	61 ± 0
			R	65 ± 1	49 ± 1	46 ± 2	74 ± 1	55 ± 1	61 ± 0
		High Leak	S	47 ± 2	32 ± 1	29 ± 0	53 ± 1	39 ± 1	54 ± 0
			O	65 ± 1	70 ± 0	46 ± 2	76 ± 1	51 ± 1	62 ± 0
			R	64 ± 2	49 ± 1	45 ± 1	74 ± 1	53 ± 1	61 ± 0

Values were measured only in synchronic cycles

### General performance

**Fig. 5:**
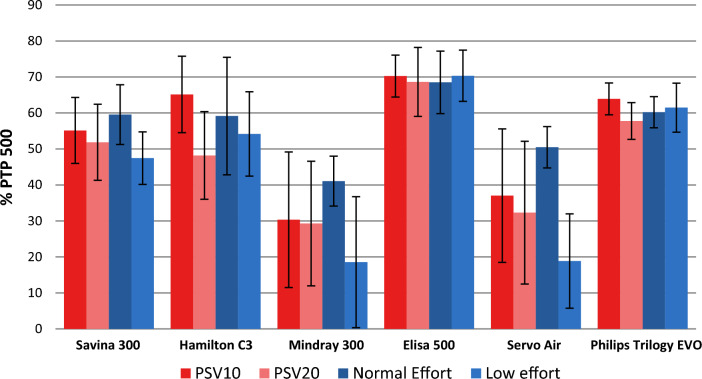
PTP500 (mean and SD in %) in PSV10 and 20 for mean values of mechanical pattern and leak level, comparing low and normal inspiratory effort

Leak level did not significantly affect PTP500 regardless mechanical pattern; changes in PSV from 10 to 20 cm H_2_O slightly decreased PTP500 (from 2 to 26%) as represented in Fig. 5. Low effort also decreased PTP500 especially for Mindray300 and Servo Air. Dräger Savina 300, Hamilton C3, Elisa 500 and Philips Trilogy Evo/EV300 obtained mean PTP500 > 50% in almost all conditions. Mean values did not change for Elisa 500, which showed the most homogeneous values in all conditions followed by Philips Trilogy Evo/EV300. Mindray 300 and Servo Air comparatively developed the worst pressurization capacity in all conditions.

**Fig. 6:**
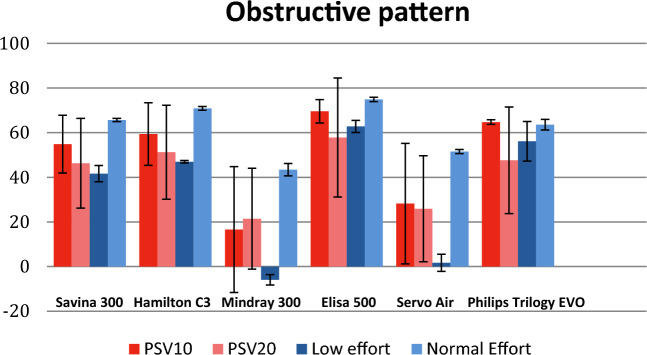
PTP500 (in %) in PSV10 and PSV20 for obstructive mechanical pattern with high leak, comparing low and normal inspiratory effort

**Fig. 7:**
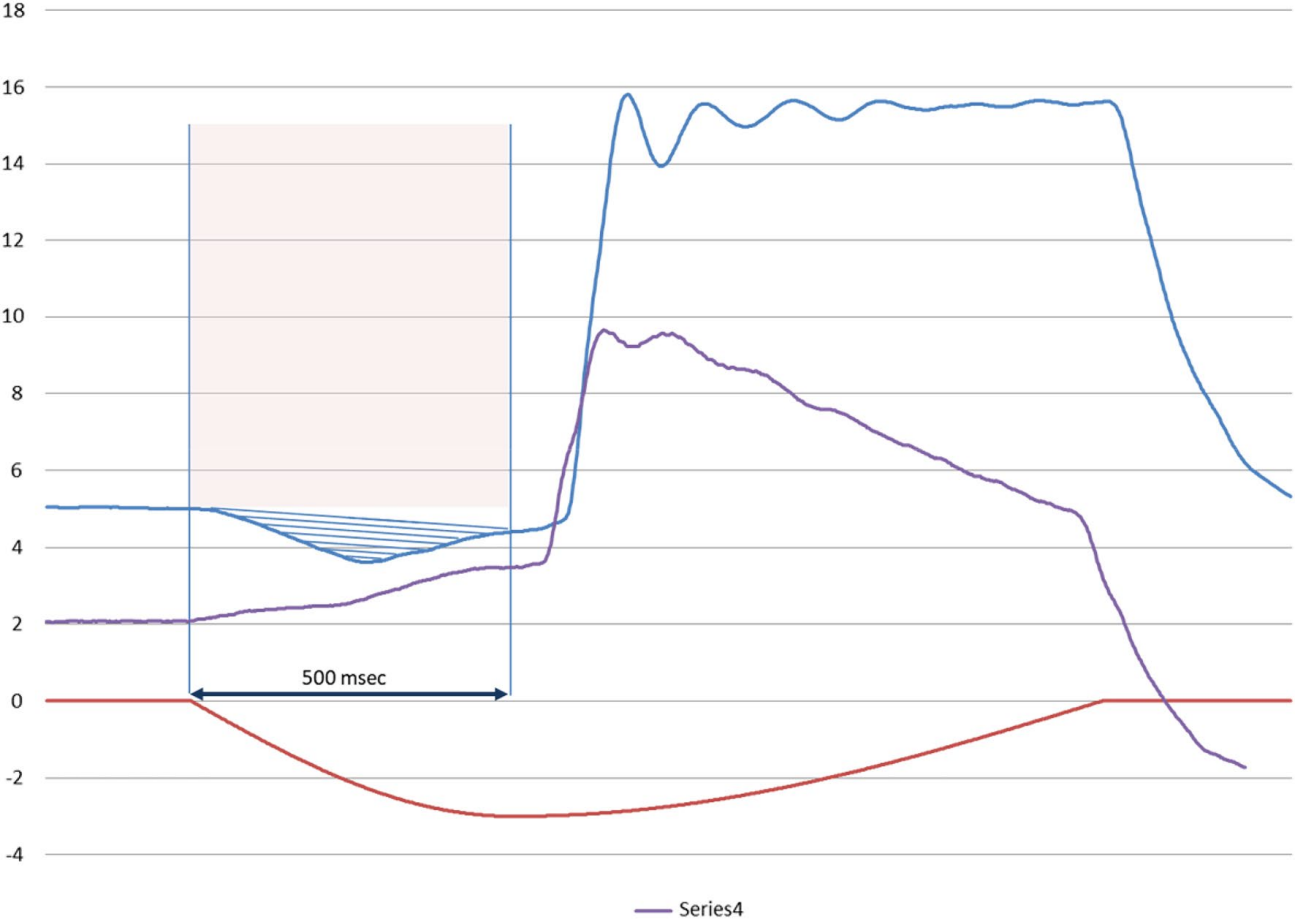
Mindray 300 during PSV 10 with obstructive pattern, low effort and moderate leak. Time delay from drop in Paw until rising in flow (in purple) and Paw (in blue) is longer than 500 ms and makes the area under airway pressure in the first 500 ms to become negative (striped area). Ideal PTP500 is represented by the shaded area

As recorded in Table 4 and represented in Fig. 6, the worst pressurization capacity was developed under obstructive mechanical pattern and low effort in Mindray 300 and Servo Air. These worrying results, as obstructive patients are frequent users of home care therapies, may be explained by the interdependence of PTP500 and TDT. Negative values of PTP500 (Mindray 300) indicate that trigger delay time is longer than 500 ms. This way the area under Paw curve in the first 500 ms become negative (see Fig. 7). If TDT was lower, Paw rise would occur sooner and PTP500 results would improve.

**Fig. 8:**
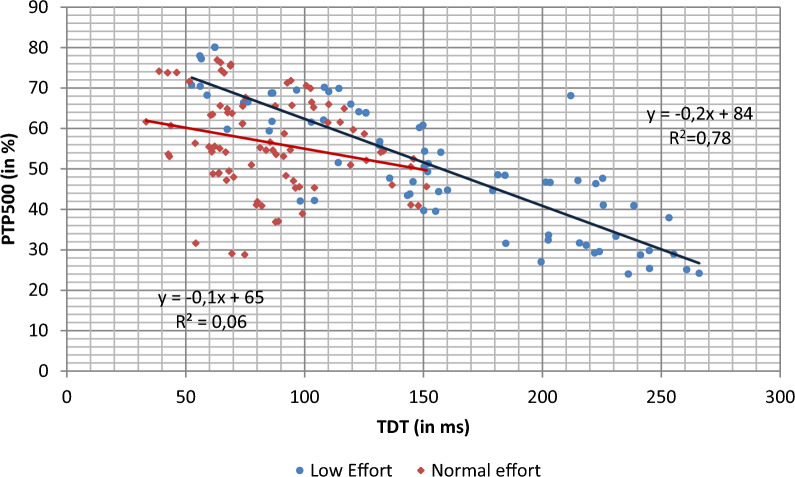
Influence of TDT in pressurization capacity (PTP500) comparing low and normal effort; dots represent average values of different conditions (PSV, mechanical pattern and leak level)

In order to elucidate why such differences in PTP500 results were obtained, influence of trigger delay (TDT) over PTP500 in our bench test was analyzed; Fig. 8 shows the correlation between PTP500 and TDT. As seen, the relationship depends on the effort level: a clear relationship can be seen for low effort conditions. However, this correlation disappears for values obtained under conditions of normal effort. Inverse relationship of TDT-PTP500 may explain the worse results for Mindray 300 producing negative PTP500 values while obstructive pattern under low effort (see Fig. 6).

### Asynchrony index

**Fig. 9:**
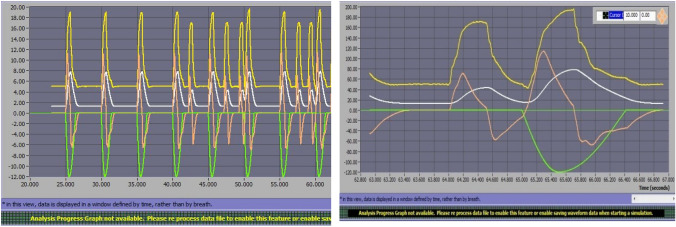
Paw in yellow, Volume in white, Flow in red and Pressure muscular in green: ELISA 500 in condition 24 (PSV 10, Normal effort, high leak). As shown, there is no inspiratory effort prior to the first airway pressure increase (AT); in contrast, the second cycle starts with the inspiratory effort

**Fig. 10:**
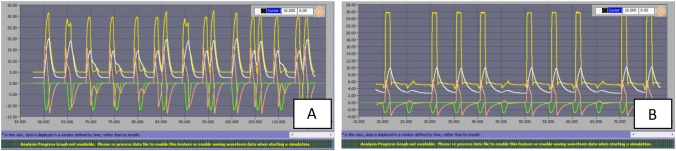
**A** 12 cycles of Elisa 500 in condition 31 recorded with AT and double trigger, **B** several IE with Mindray 300 in condition 26. Many inspiratory efforts (in green) are not followed by airway pressure (in yellow) or flow (in red) increase. Condition nomenclature in Table 1

**Fig. 11:**
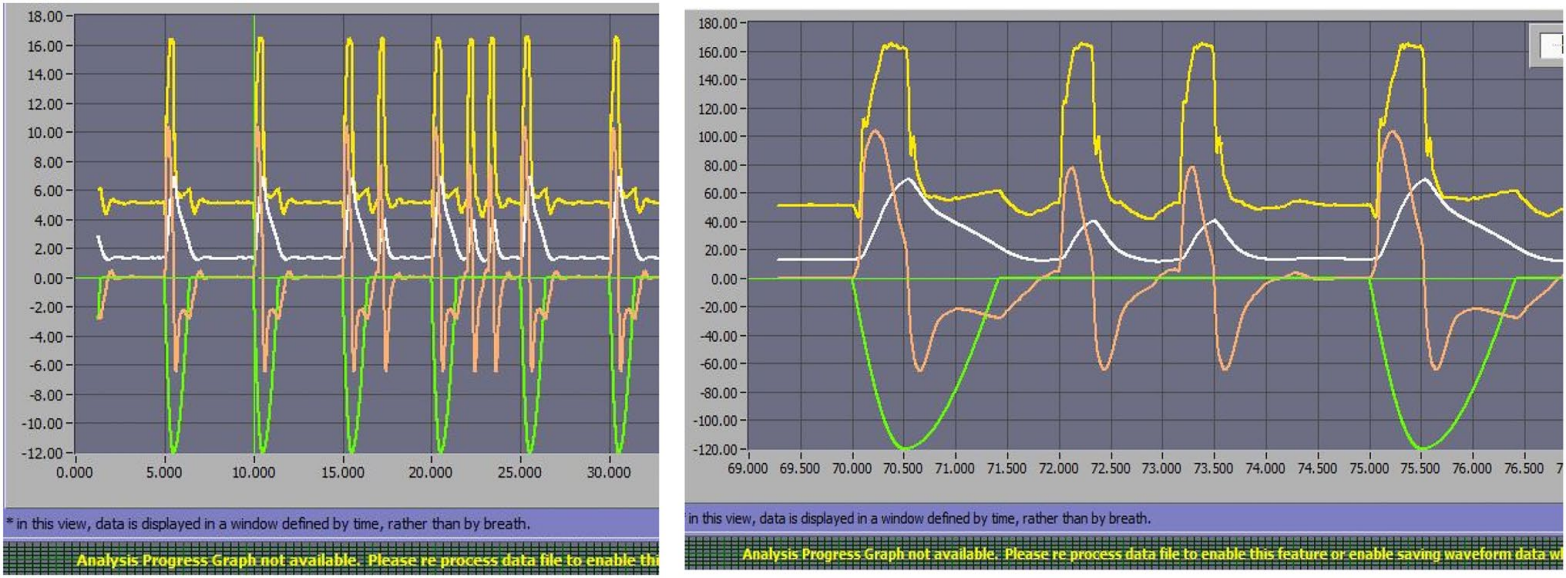
AT developed by Hamilton C3 during PSV10 in conditions 21 and 24 (restrictive pattern, normal effort and moderate and high leak, respectively). AT appeared even after modifying trigger settings (Trigger sensitivity 1 lpm)

Synchronization assessment of each ventilator is of paramount importance at clinical level for patient´s comfort and because asynchronies can determine the failure of mechanical ventilation with potential deleterious consequences. In general, few asynchronies developed during this analysis (Table 5). Overall incidence was 12% (21asynchronies out of 180 conditions); 13% during PSV conditions (18 out of 144 conditions) and 8% during ACV (3 out of 36 conditions). The most frequent ones were Auto-Triggering (AT) and Double-Trigger (DT) (described in Table 6). Remarkably Philips Trilogy Evo/EV300 did not develop any time-asynchrony under the analyzed conditions. Elisa 500 showed asynchronies (AT) in pressure support at almost any condition (Figs. 9 and 10), increasing in PSV20 regardless changes in leak level and muscle effort (Table 6). Savina 300 showed only two asynchronies: DT in ACV and Ineffective Effort (IE) in PSV20. Hamilton C3 developed few AT and DT with restrictive mechanical pattern (Fig. 11).

**Table 5:** Asynchrony index (%) for the analyzed ventilators in all conditions

Mode	Conditions		Asynchrony Index (%) for the analyzed ventilators					
			Mechanical pattern: Standard/Obstructive/Restrictive					
	Effort	Leak	Dräger Savina 300	Elisa 500	Hamilton C3	Servo Air	Mindray 300	Philips Trilogy Evo
ACV	Low	Moderate	0/0/38		0/0/0			0/0/0
		High	0/0/0		0/0/17			0/0/0
	Normal	Moderate	0/0/0		18/0/0			0/0/0
		High	0/0/0		0/0/0			0/0/0
PSV10	Low	Moderate	0/0/0	0/0/17	0/0/0	0/0/17	0/0/0	0/0/0
		High	0/0/0	0/0/0	0/0/0	0/0/0	0/0/0	0/0/0
	Normal	Moderate	0/0/0	8/0/0	0/0/25	17/0/0	0/0/0	0/0/0
		High	0/0/0	41/0/58	0/0/33	0/0/0	0/0/0	0/0/0
PSV20	Low	Moderate	0/8/0	69/0/68	0/0/0	0/0/0	0/25/0	0/0/0
		High	0/0/0	23/0/65	0/0/0	0/8/0	0/33/0	0/0/0
	Normal	Moderate	0/0/0	81/0/80	0/0/0	0/0/8	8/0/0	0/0/0
		High	0/0/0	23/0/62	0/0/8	0/0/0	0/0/0	0/0/0

**Table 6:** Types and incidence of asynchronies developed by each ventilator in all conditions analyzed

Ventilator	Asynchronies	Incidence in conditions	Total incidence (%)
Savina 300	Double trigger	ACV: 3	3
	Ineffective effort	PSV: 26	3
Hamilton C3	Double trigger	ACV: 6	3
	Auto-triggering	ACV: 6, 7	6
		PSV: 21, 24, 36	8
Mindray 300	Ineffective effort	PSV: 26, 29	8
Elisa 500	Auto-triggering	PSV: 15,18,19,24,25,27,28,30,31,33,36	46
Servo Air	Ineffective effort	PSV: 29	4
Philips Trilogy Evo			0

Condition nomenclature in Table 1

It is important to describe the asynchronies developed by the analyzed ventilators as they play an important role during NIV: primarily because asynchronies directly affect comfort and tolerance of mechanical ventilation [1], and secondly as they are essential for protective mechanical ventilation as has been recently highlighted. [34].

AT and DT were the most frequently developed asynchronies, which are directly related to patient comfort because they are un-demanded pressure cycles. AT developed even with normal effort and pressure support levels used in clinical practice. In contrast, few ineffective efforts developed, which also affect comfort due to the lack of support.

All these asynchronies are highly dependent on trigger sensitivity, already mentioned as it was adjusted during the experiments to avoid AT. In our performance analysis, whenever we detected AT in any condition, we modified trigger sensitivity up to 1.6 lpm (for Servo Air) to cope with them. In many cases, asynchronies mentioned developed in identical conditions for many devices, so they must be linked to performance characteristics of the equipment itself. Ventilators´ intrinsic behavior that favors asynchronies could be related to the magnitude of bias flow (set by each manufacturer). Flow sensitivity should be adjusted around 1/2 of bias flow magnitude; this way high bias flow will require a higher flow sensitivity which may prolong TDT, decrease PTP500 and favor asynchronies.

## Discussion

As different ventilators have been recently marketed, it is important to know how they perform in order to choose the device which best fits each patient needs. This study explored general performance of six ventilators using the ASL5000^©^ lung simulator, validated in previous analysis [8, 9, 35, 36]. They were tested during NIV against different combinations of mechanical pattern, ventilatory mode, leak level and inspiratory effort, with similar methodology as our previous work [32]. Measured results included volume delivered, time response to trigger, pressurization capacity and synchronization. As far as we know, our publication is one of the few analyzing VT in ACV during NIV [37, 38]. Despite doubts about leak compensation from portable ventilators discussed in previous publications [23, 36, 39, 40], the analyzed devices successfully compensated changeable air leaks. Variable efforts in NIV have not been as addressed in recent literature, but we also found a good compensation for effort variations with our devices. The availability of ACV mode for NIV in only three devices and the interdependence between PTP500 and TDT values with low effort were new findings.

Main results:

1. ACV mode for NIV was available in three out of six ventilators analyzed: Savina 300, Hamilton C3 and Philips Trilogy Evo/EV300. All of them obtained V_T_ within the security range in all conditions, so they could be reliable devices for home use under ACV even with variable leaks or inspiratory efforts.
2. Trigger delay (TDT) remained into an acceptable range < 200 ms for many conditions in spite of PSV level and leak magnitude changes, as already seen in previous series [28]. In contrast, TDT worsened significantly with low muscle effort, even doubling TDT values except for Philips Trilogy Evo/EV300. Servo Air and Mindray 300 obtained the highest TDT values even after trigger sensitivity adjustment.
3. Results of pressurization were worse than expected for NIV. PTP500 remained unaffected regardless leak level. Increased pressure support and lower effort slightly decreased PTP500 results for Mindray 300 and Servo Air under obstructive lungs and low effort. Elisa 500 and Philips Trilogy Evo/EV300 achieved similar PTP500 values for all conditions and showed good performance above 50% in all conditions with normal effort.
4. The effect of TDT over PTP500 was isolated and we found that under low effort, PTP500 presented interdependence with TDT (lower pressurization with higher TDT values). That is why Mindray 300 showed inability for airway pressurization in the first 500 ms even if, after that time interval, pressurization was correct.
5. In general, Asynchrony Index was low. AT and DT were the most frequently developed asynchronies. To avoid them, trigger sensitivity was adjusted but in some cases AT did not disappear. That could be explained by performance characteristics of the equipments (ventilators´ intrinsic behavior). Philips Trilogy Evo/EV300 did not show any asynchrony with trigger sensitivity of 1 lpm.

**Fig. 12:**
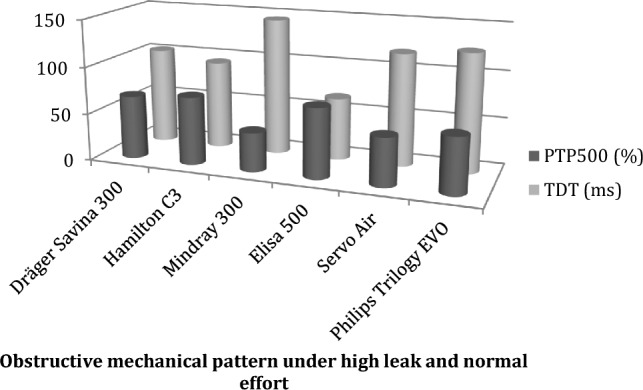
Graphic representation of TDT and PTP500 measured for the six ventilators analyzed in the obstructive most frequent home-care condition

All ventilators demonstrated great capability to counteract the effect of higher leaks, as performance did not worsen in those conditions. This fact points out the great technological improvements done in the last years, as previous assessments achieved heterogeneous results [36, 41]. In contrast, differences observed with changes in muscle inspiratory effort must be taken into account for ventilator choice (for example, patients with neuromuscular weakness must be carefully assessed after initiating ventilatory therapy to avoid hypoventilation and discomfort [42, 43]). Pressurization capacity was highly dependent on TDT values, so both must be checked and analyzed together (Table 7). Elisa 500 showed the best results of trigger response and pressurization capacity (Fig. 12).

**Table 7:** Values for obstructive mechanical pattern under high leak and moderate effort (condition 35), linked to Fig. 12

Obstructive mechanical pattern under high leak and moderate effort PSV20	TDT (in ms)	PTP500 (in %)
Dräger Savina 300	103	67
Hamilton C3	94	72
Mindray 300	145	41
Elisa 500	66	74
Servo Air	119	51
Philips Trilogy Evo/EV300	88	62

*TDT* trigger delay time, %*PTP500* percent of ideal pressure time product in the first 500 ms

### Clinical implications

The perfect device for NIV does not exist because we must take into account characteristics of paramount importance and the variable clinical situation of each patient. A good approach on the ideal device for NIV is the ventilator that combines security (safe V_T_ and high PTP500) and comfort (low TDT and synchronization); it should compensate variable leaks and adapt to different inspiratory efforts regardless level of support without developing asynchronies. Even if we did not find the perfect device, some are close to the best performance and almost all worked within acceptable values.

Whenever ACV in NIV is needed, Savina 300, Hamilton C3 and Philips Trilogy Evo/EV300 are good options. During PSV, some differences could make a difference on device selection. For patients with low muscle effort, Philips Trilogy Evo/EV300 developed a fast response without asynchronies, and in case of normal effort both Trilogy Evo/EV300 and Elisa 500 presented similar performance and could be interchangeable. Worse options for patients which need a fast response are Servo Air and Mindray 300, as they presented longer TDT even after trigger sensitivity adjustment. For patients whose main concern is airway pressurization, Elisa 500 achieved the best performance even in adverse conditions; in contrast, Mindray 300 is the last option because of the worst PTP500 linked to TDT influence. As tolerance during home care is matter of great importance, good options seem both Philips Trilogy Evo/EV300 and Savina300, because of their lower rates of asynchronies combined with faster TDT responses, specially the first one. Also, they both have ACV mode so neuromuscular patients can benefit from these options, as whenever they need controlled ventilation at home, worse adaptation leads to lower survival rates and poor prognosis [42].

### Limitations

Our findings should be interpreted in the context of several limitations. Firstly, this was a laboratory analysis, thus clinical studies with patients could show different results due to variable conditions associated to patients´ heterogeneous characteristics and dynamic changes. Secondly, we chose thirty-six conditions that only reproduced few clinical scenarios even if more complex conditions could be taken into consideration. Performance in ACV mode was analyzed even if it does not have a spread clinical use. We decided to consider it because some ventilators offer this mode and it has its indication in, for instance, patients with neuromuscular pathology. Thirdly, many ventilator settings, as trigger sensitivity, can be adjusted in different ways during the clinical practice. In contrast, we are limited in the context of using a lung model. Also, by using ASL5000^©^the spontaneous inspiratory profile is not modified by pressurization during the inspiratory phase. Errors in the measurements during the experiment may have occurred but being systematic they would affect all the devices in a similar way. At last, we evaluated the ventilators based in four parameters that we considered to be representative of their performance, and that have been already used in previous publications [9, 17, 44, 45]. Other combinations and setting would generate different results.

## Conclusions

As a result of the bench test developed, we found a relevant variation in the performance among devices. In PSV conditions, Philips Trilogy Evo/EV300, Hamilton C3 and Elisa 500 seemed the most reliable devices, as they showed the best performance in terms of trigger response and pressurization capacity. When patient comfort was of main concern, only Philips Trilogy Evo/EV300 did not develop any time-asynchrony and must be the preferable choice; Dräger Savina 300 and Servo Air also seem good options. In contrast, Elisa 500 showed poor synchronization. In case ACV is necessary, only three well founded options remain available: Dräger Savina 300, Hamilton C3 and Philips Trilogy Evo/EV300.

### Supplementary Information

Below is the link to the electronic supplementary material.

Supplementary file1 (DOCX 262 KB)

## Data Availability

All data generated or analyzed during this study are included in this published article (and its supplementary information files). Data are however available from the corresponding author upon reasonable request in saradacuris@hotmail.com or anestesia.saramartinezcastro@gmail.com.

## Abbreviations

ACV: assist-control ventilation
AI: asynchrony index
ASL500^®^: Active Servo Lung 5000
AT: auto-triggering
Bpm: breaths per minute
BTPS: body temperature pressure saturated
Crs: compliance of the respiratory system
DT: double trigger
E: effort
H: high leak level
ICU: intensive care unit
IE: ineffective effort
I/E: inspiration/expiration
Le: low effort
Lpm: liters per minute
M: moderate leak level
Ne: normal effort
NIV: non-invasive ventilation
O: obstructive mechanical pattern
P_0.1_: airway occlusion pressure in the first 100 ms
Paw: airway pressure
PEEP: positive end-expiratory pressure
Pmus: muscle pressure
PSV: pressure support ventilation
PTP500: pressure–time product in the first 500 ms
R: restrictive mechanical pattern
Raw: airway resistance
RR: respiratory rate
RT: reverse or double trigger
S: standard mechanical pattern
S: seconds
TDT: trigger delay time
VT: tidal volume
