# Supplementary material for: Are all ventilators for NIV performing the same? A bench analysis
Source: J Clin Monit Comput. 2023 Jul 31;37(6):1497–511. doi: 10.1007/s10877-023-01019-z (PMC10651552; doi:10.1007/s10877-023-01019-z)
Supplement: Supplementary file 1 — Supplementary file1 (DOCX 262 KB) [file 10877_2023_1019_MOESM1_ESM.docx]

**SUPLEMENTARY MATERIAL**

**Occlusion maneuver**

| Inspiratory effort | Paw measurement (cmH_2_O) |
| --- | --- |
| Low (-0,9 cmH_2_O) | 0,86 ± 0,01 |
| Moderate (-3,5 cmH_2_O) | 3,34 ± 0,02 |

Table 1 Measurement of airway pressure after low and moderate inspiratory effort with an occlusion maneuver in the inspiratory port of the lung model. Paw: airway pressure.

After the occlusion maneuver, the following graphs were obtained:

1. Low inspiratory effort:


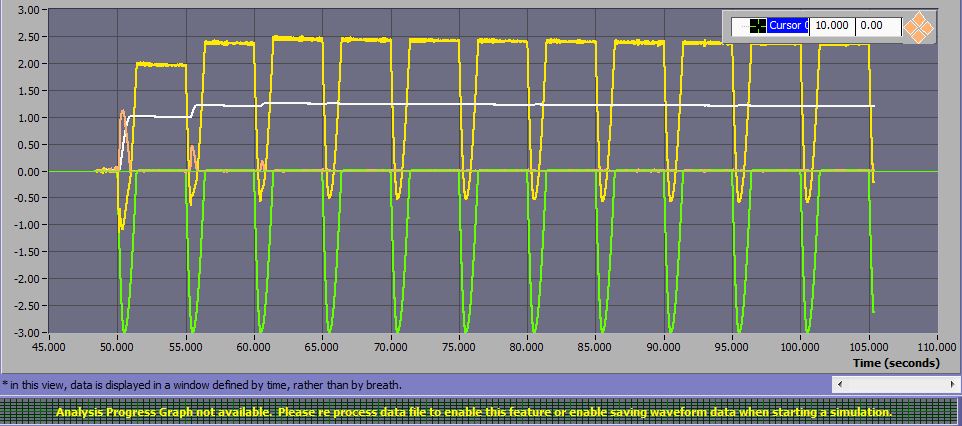

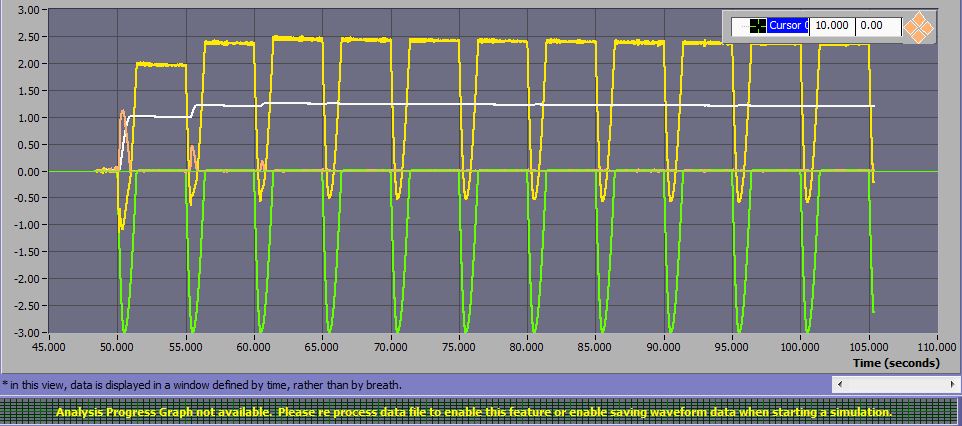


Figure 1 Occlusion maneuver view in ASL5000® program under low inspiratory effort.Green line: muscle inspiratory effort (pressure drop), yellow line: airway pressure.

Figure 2 Graphic representation of the occlusion maneuver under low inspiratory effort.Red line: muscle inspiratory effort, blue line: airway pressure.

1. Moderate inspiratory effort:


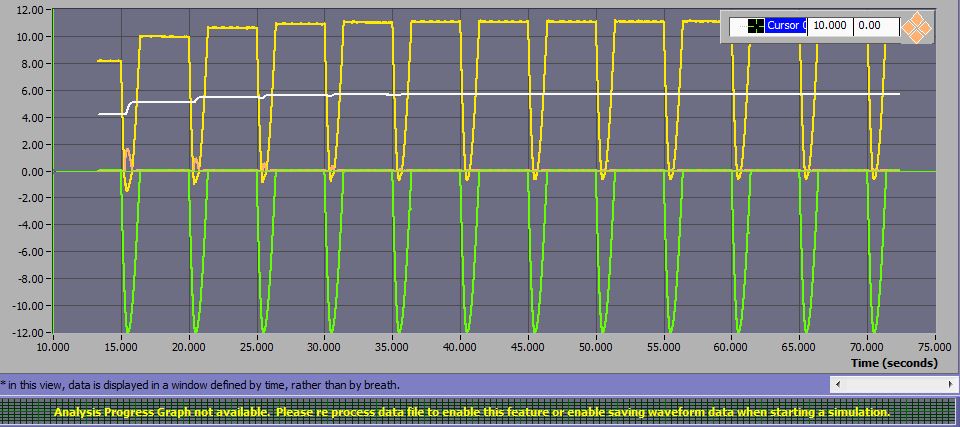

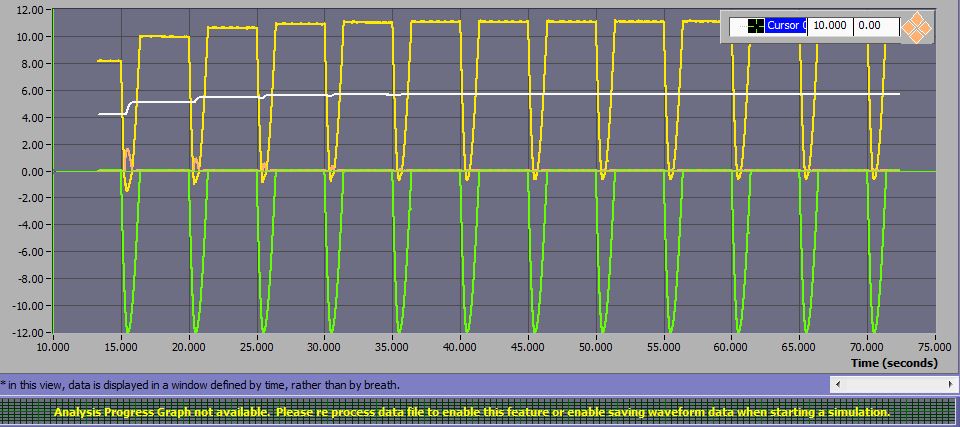


Figure 3 Occlusion maneuver view in ASL5000® program under moderate inspiratory effort. Green line: muscle inspiratory effort (pressure drop), yellow line: airway pressure.

Figure 4 Graphic representation of the occlusion maneuver under moderate inspiratory effort. Red line: muscle inspiratory effort, blue line: airway pressure.

Table 2 Mean Trigger delay time (TDT, in ms) and standard deviation (SD) in all conditions for the ventilators analyzed.

|  | **DrägerSavina 300** | | **Hamilton C3** | | **Mindray 300** | | **Elisa 500** | | **Servo Air** | | **Philips Trilogy Evo** | |
| --- | --- | --- | --- | --- | --- | --- | --- | --- | --- | --- | --- | --- |
|  | **Mean** | **SD** | **Mean** | **SD** | **Mean** | **SD** | **Mean** | **SD** | **Mean** | **SD** | **Mean** | **SD** |
| **Condition 1** | 164,17 | 16,89 | 94,33 | 13,17 | 76,18 | 9,9 |  |  | 129,83 | 19,97 | 60 | 9,59 |
| **Condition 2** | 141,33 | 13,41 | 236 | 62,88 | 235,33 | 9,96 |  |  | 218,36 | 12,16 | 128,25 | 6,8 |
| **Condition 3** | 167,75 | 22,1 | 73,17 | 13,89 | 115,83 | 11,98 |  |  | 252,2 | 13,71 | 63,56 | 10,19 |
| **Condition 4** | 145 | 17,47 | 89,09 | 17,92 | 83,45 | 12,07 |  |  | 220,8 | 33,36 | 73,25 | 4,53 |
| **Condition 5** | 305,83 | 16,03 | 252,18 | 20,09 | 230 | 20,32 |  |  |  |  | 219,6 | 10,49 |
| **Condition 6** | 150 | 18,25 | 99,2 | 6,26 | 115,82 | 11,68 |  |  |  |  | 85,83 | 7,51 |
| **Condition 7** | 74 | 11,15 | 55,11 | 6,17 | 68,86 | 15,61 |  |  | 112,83 | 4,63 | 75,4 | 3,89 |
| **Condition 8** | 128,17 | 48,34 | 87,45 | 4,01 | 99,17 | 6,29 |  |  | 213,83 | 6,29 | 104,8 | 6,55 |
| **Condition 9** | 77 | 4,47 | 49,2 | 5,93 | 78,17 | 15,29 |  |  | 125,33 | 9,45 | 63,14 | 5,76 |
| **Condition 10** | 66 | 7,82 | 43,82 | 4,77 | 83,56 | 6,06 |  |  | 229,33 | 13,32 | 71,4 | 4,43 |
| **Condition 11** | 138,91 | 15,42 | 85,2 | 5,09 | 107,33 | 3,75 |  |  | 200 | 12,65 | 112,5 | 8,54 |
| **Condition 12** | 75,67 | 4,25 | 55,17 | 4,71 | 81,71 | 9,62 |  |  |  |  | 73,75 | 2,92 |
| **Condition 13** | 151,83 | 13,92 | 96,67 | 10,42 | 202,5 | 16,54 | 52,5 | 2,84 | 202,67 | 19,09 | 74,33 | 4,08 |
| **Condition 14** | 203,33 | 12,4 | 225,4 | 7,89 | 512,33 | 49,28 | 119,5 | 11,79 | 400,5 | 22,55 | 125,82 | 6,9 |
| **Condition 15** | 108 | 11,25 | 114,5 | 13,89 | 231 | 13,6 | 56,6 | 4,53 | 241,33 | 22,09 | 86 | 5,12 |
| **Condition 16** | 145,67 | 22,3 | 110,22 | 7,1 | 215,67 | 8,73 | 56,17 | 5,49 | 224 | 16,86 | 76,18 | 3,74 |
| **Condition 17** | 225,67 | 19,83 | 201,45 | 15,13 | 542,83 | 38,61 | 122,83 | 13 | 417 | 35,5 | 125,83 | 10 |
| **Condition 18** | 131,67 | 24,45 | 108,33 | 12,35 | 245 | 17,57 | 56 | 12,52 | 255,33 | 10,07 | 86,67 | 7,05 |
| **Condition 19** | 68,17 | 11,98 | 54 | 3,62 | 87,83 | 4,13 | 43,8 | 3,05 | 92,2 | 4,66 | 59,83 | 6,29 |
| **Condition 20** | 103,83 | 6,41 | 92,73 | 8,78 | 147,83 | 19,27 | 65 | 3,77 | 126 | 6,32 | 94,67 | 5,87 |
| **Condition 21** | 64,5 | 5,13 | 64,67 | 2 | 77,67 | 12,15 | 38,8 | 3,29 | 91,5 | 5,79 | 60,5 | 4,98 |
| **Condition 22** | 70,17 | 11,49 | 62,18 | 6,66 | 89 | 7,93 | 33,33 | 6,63 | 95,33 | 7,1 | 64,33 | 4,74 |
| **Condition 23** | 103 | 9,78 | 94,22 | 4,06 | 144,67 | 6,29 | 66,17 | 4,39 | 119,33 | 8,88 | 87,67 | 4,25 |
| **Condition 24** | 67,67 | 6,87 | 63,25 | 2,82 | 104,17 | 10,21 | 51,56 | 21,63 | 85,5 | 6,88 | 61,33 | 7,92 |
| **Condition 25** | 144,33 | 17,12 | 98,17 | 7,88 | 184,67 | 9,35 | 212 | 47,03 | 245,11 | 17 | 85,17 | 6,9 |
| **Condition 26** | 253,33 | 15,22 | 215 | 15,09 | 582,67 | 65,88 | 148,33 | 18,23 | 461,5 | 33,89 | 181,33 | 15,73 |
| **Condition27** | 131,5 | 13,27 | 135,83 | 26,29 | 218,5 | 23,42 | 62,33 | 2,94 | 260,73 | 9,73 | 86,33 | 10,88 |
| **Condition 28** | 143,33 | 40,69 | 104,22 | 5,78 | 199,5 | 20,43 | 59 | 4,03 | 236,17 | 35,03 | 67,5 | 12,6 |
| **Condition 29** | 238,5 | 13,19 | 222,5 | 16,62 | 584 | 50,14 | 149,83 | 15,9 | 486,4 | 37,59 | 184,33 | 14,91 |
| **Condition 30** | 179,17 | 36,5 | 114,17 | 18,67 | 222 | 11,41 | 62,29 | 4,23 | 266 | 7,59 | 102,83 | 9,16 |
| **Condition 31** | 61,5 | 6,83 | 59,45 | 4,11 | 69,45 | 5,8 | 42,67 | 1,15 | 80,18 | 16,28 | 61 | 4,13 |
| **Condition 32** | 110,17 | 4,63 | 100,73 | 4,58 | 151,33 | 10,49 | 68,83 | 5,94 | 145,83 | 5,56 | 109,83 | 6,79 |
| **Condition 33** | 67,5 | 5,6 | 63,83 | 4,86 | 97,83 | 9,81 | 42,4 | 4,98 | 94 | 5,93 | 73,83 | 7,06 |
| **Condition 34** | 67,17 | 8,2 | 54,25 | 3,62 | 74,83 | 8,33 | 43,2 | 4,54 | 99,09 | 6,16 | 66,83 | 5,15 |
| **Condition 35** | 116,67 | 4,12 | 102,55 | 7,1 | 136,83 | 10,43 | 69 | 4,63 | 144,67 | 5 | 115 | 7,6 |
| **Condition 36** | 69,5 | 7,44 | 64 | 6,45 | 96,17 | 9,04 | 46,25 | 4,95 | 91,17 | 10,36 | 74 | 8,05 |
